# Multiphase CT angiography perfusion maps for predicting target mismatch and ischemic lesion volumes

**DOI:** 10.1038/s41598-023-48832-9

**Published:** 2023-12-11

**Authors:** Kevin J. Chung, Sachin K. Pandey, Alexander V. Khaw, Ting-Yim Lee

**Affiliations:** 1 Department of Medical Biophysics, The University of Western Ontario, London, ON Canada; 2 Robarts Research Institute and Lawson Health Research Institute, London, ON Canada; 3 Department of Medical Imaging, The University of Western Ontario, RRI 1200D, 1151 Richmond Street N, London, ON N6A 5B7 Canada; 4 Department of Clinical Neurological Sciences, The University of Western Ontario, London, ON Canada

**Keywords:** Stroke, Imaging techniques, Biomedical engineering

## Abstract

The complexity of CT perfusion (CTP) acquisition protocols may limit the availability of target mismatch assessment at resource-limited hospitals. We compared CTP mismatch with a mismatch surrogate generated from a simplified dynamic imaging sequence comprising widely available non-contrast CT (NCCT) and multiphase CT angiography (mCTA). Consecutive patients with anterior circulation acute ischemic stroke who received NCCT, mCTA, and CTP were retrospectively included in this study. An mCTA-perfusion (mCTA-P) dynamic series was formed by co-registering NCCT and mCTA. We simulated an ideal mCTA-P study by down-sampling CTP (dCTP) dynamic images according to mCTA timing. Ischemic core and penumbra volumes were estimated by cerebral blood flow and Tmax thresholding, respectively, on perfusion maps calculated independently for CTP, dCTP, and mCTA-P by deconvolution. Concordance in target mismatch (core < 70 ml, penumbra ≥ 15 ml, mismatch ratio ≥ 1.8) determination by dCTP and mCTA-P versus CTP was assessed. Of sixty-one included patients, forty-six had a CTP target mismatch. Concordance with CTP profiles was 90% and 82% for dCTP and mCTA-P, respectively. Lower mCTA-P concordance was likely from differences in collimation width between NCCT and mCTA, which worsened perfusion map quality due to a CT number shift at mCTA. Moderate diagnostic agreement between CTP and mCTA-P was found and may improve with optimal mCTA scan parameter selection as simulated by dCTP. mCTA-P may be a pragmatic alternative where CTP is unavailable or the risks of additional radiation dose, contrast injections, and treatment delays outweigh the potential benefit of a separate CTP scan.

## Introduction

CT perfusion (CTP)-based mismatch, defined as a disparity between ischemic core volume and a penumbral criterion, was an imaging-based selection criteria investigated for late-window endovascular thrombectomy (EVT) in patients with anterior circulation large vessel ischemic stroke. The DAWN trial used a clinical-core mismatch to enrol patients for EVT between 6 to 24 h after stroke onset if perfusion imaging-based ischemic core volume was small relative to clinical deficit as determined by the National Institutes of Health Stroke Scale (NIHSS)^1^. Similarly, the DEFUSE-3 trial used a core-penumbra volume mismatch to select patients for late-window EVT if the ischemic core volume was small relative to the penumbral volume determined by automated perfusion imaging software^2^. Core-penumbra mismatch has also been demonstrated for appropriate patient selection in the early EVT time window (< 6 h after stroke onset)^3^ as well as for thrombolysis 4.5–9 h after stroke onset^4^.

The utilization and availability of CTP remains relatively low whereas non-contrast CT (NCCT) and CT angiography (CTA) are widely utilized in patients presenting with acute ischemic stroke^5,6^. In a retrospective study of advanced acute stroke imaging utilization in Texas, Kim et al. found that only 14% of Texas hospitals, most of which were urban academic centres, were capable of CTP imaging whereas 77% of Texas hospitals were CTA-capable^7^. While both CTA and CTP utilization are increasing^5–7^ after the landmark late-window EVT trials^1,2^, CTP is still expected to be less accessible than CTA in part due to a lack of emergent availability of trained CT technologists for routine acquisition, hesitancy due to additional radiation dose, contrast injections, scan time, and other logistical considerations. Automated assessment of treatment selection criteria, such as perfusion mismatch, with standard acute stroke CT is therefore highly desirable. Perfusion mismatch derived from standard acute stroke CT would require less expertise in scan acquisition compared to a dedicated CTP study, less imaging time, and less radiation exposure.

Multiphase CTA (mCTA) is a simple extension to a routine acute stroke head-neck CTA that adds two delayed intracranial CTA images^8^. In many parts of Canada, NCCT and mCTA are considered standard neuroimaging for all patients presenting with a suspected acute ischemic stroke^9^. In principle, mCTA is similar to CTP as they are both dynamic, time-resolved contrast-enhanced imaging techniques, but mCTA has poorer temporal resolution compared to CTP. In this study, we investigated the feasibility of predicting perfusion mismatch from NCCT and mCTA-derived perfusion maps (multiphase CT angiography-perfusion, mCTA-P) and compared diagnostic agreement with CTP. Specifically, this study focused on simplifying perfusion imaging scan protocols with mCTA-P, potentially improving the accessibility of this decision support tool.

## Methods

### Study participants

Consecutive patients presenting to our institution with a suspected acute stroke between May 2021 to September 2021 were identified for this retrospective study. Inclusion criteria were (1) patients with a confirmed anterior circulation stroke and (2) received CT imaging according to our institution’s hyperacute stroke protocol, which comprised of an NCCT, mCTA, and CTP. Exclusion criteria were (1) incomplete hyperacute stroke CT protocol, (2) stroke with intracranial hemorrhage at admission, (3) technically inadequate scan including severe patient movement or CT artifacts preventing reliable calculation of perfusion maps, or (4) absence of an anterior circulation arterial occlusion. Patient demographics, clinical characteristics, site of occlusion, and treatment modality were obtained from clinical notes. This study was conducted in accordance with institutional guidelines and regulations and was approved by the Western University Health Science Research Ethics Board. Informed consent was waived by the Western University Health Science Research Ethics Board for this retrospective study of standard of care CT images.

### Imaging protocol

CT studies were acquired on a LightSpeed VCT 64-row CT scanner (GE Healthcare, Waukesha, WI). Scan parameters are summarized in Table 1. NCCT acquired 14 cm whole brain coverage using a shuttle mode acquisition (step-and-shoot; seven 2 cm slabs). Multiphase CTA then followed with a helical head-neck CTA acquired at peak enhancement of the normal brain arteries then two additional delayed intracranial CTA images at 8 s intervals^8^. Eighty ml of iodinated contrast material (Optiray 320; Mallinckrodt Pharmaceuticals, St. Louis, MO) was injected at 4.5 ml/s followed by a 60 ml saline chase at the same rate. Peak arterial enhancement in the normal brain was timed by a test bolus method with monitoring at the common carotid artery. CTP was acquired after mCTA with a 40 ml injection of the same contrast material, injection rate, and saline chase. After a 5 s prep delay, 22 dynamic images were acquired at 2.8 s interval followed by 6 additional images at 15 s interval. The bow tie filter and reconstruction kernel were consistent between the three CT studies. Dose-length products were approximately ≈ 600 mGy·cm for NCCT, ≈ 1300 mGy·cm for the head-neck CTA and ≈ 260 mGy·cm for each of the two delayed intracranial CTA phases, and ≈ 875 mGy·cm for CTP.

**Table 1:** Hyperacute stroke CT scan parameters.

Parameter	Non-contrast CT	CT angiography	CT perfusion
Tube voltage [kV]	120	120	80
Tube current [mA]	125	~ 130 intracranial	190
Exposure time [s]	2	0.6	0.4
Beam collimation width [mm]	20	40	40
Axial coverage [cm]	14	15 intracranial	8
Scan mode	Axial (shuttle)	Helical	Axial (shuttle)
Helical pitch	N/A	0.984	N/A
Gantry tilt [°]	10 to 30	0	0
Field of view [cm]	25	25	25
Slice thickness [mm]	5, 0.625	0.625	5
Reconstruction kernel	Standard	Standard	Standard

### CT number consistency between NCCT and mCTA scan protocols

Perfusion CT assumes that the concentration of injected contrast material over time is linearly related to the CT number (density). Time-density curves (TDCs) are then used to estimate perfusion parameters via deconvolution. To correctly estimate perfusion parameters, it is therefore critical to maintain CT number consistency between dynamic contrast-enhanced CT images such that this linear relationship holds. While CTP used a fixed scan protocol between dynamic images, NCCT and mCTA used different scan parameters at our institution (Table 1; e.g., beam collimation width was 20 mm for NCCT and 40 mm for mCTA). We investigated how these differences may affect CT number consistency. Specifically, we focused on beam collimation width as it affects the level of X-ray scatter and therefore the CT number consistency between NCCT and mCTA, whereas other differences in scan parameters should not have a substantial effect^10^.

To investigate the effect of beam collimation width on CT number consistency, an anthropomorphic head phantom was scanned using a LightSpeed VCT scanner using NCCT and mCTA scan parameters. The head phantom comprised of a real human skull embedded in a Plexiglass block shaped like a human head. A series of head phantom scans were obtained to (1) compare CT number with standard NCCT and mCTA scan parameters, (2) investigate the effect of beam collimation width by scanning with a modified NCCT scan protocol at 40 mm beam collimation width instead of 20 mm while keeping other parameters the same, and (3) test a modified mCTA-P scan protocol in which beam collimation width was reduced to 20 mm to match that of NCCT. All studies were reconstructed at 5 mm slice thickness for analysis.

Head phantom images were segmented to compute mean CT number at different radial distances. Intracranial material in the head phantom was first segmented by thresholding voxels with CT numbers between 100 to 200 HU then excluding voxels outside the largest connected component. To mitigate partial volume effects near the bone interface, the segmentation was morphologically eroded by a 5 mm disk structure. The intracranial segmentation was then partitioned into 10 mm-thick rings to measure the mean CT number at different radial distances. These values were plotted against scan protocol on the horizontal axis, where each line represented a radial position. The difference in CT number between our standard NCCT and mCTA protocols in the head phantom was determined to be the CT number bias between NCCT and mCTA.

### Multiphase CTA-perfusion dynamic study

The mCTA-P dynamic study was formed by rigid and affine registration of the thin-slice NCCT with the thin-slice mCTA (BRAINS, 3D Slicer Version 4.11). To improve image signal-to-noise ratio (SNR), thin-slice mCTA-P images were axially averaged to form 10 mm-thick overlapping slices at 5 mm slice intervals. This study was then rigidly registered to the CTP average map (average of the dynamic CTP images) to facilitate analysis on the same space. Based on empirical observations of baseline to arterial peak interval times from independent CTP studies, the interval time between NCCT and the first phase of CTA was fixed at 16 s. Due to differences in beam collimation width settings between NCCT and mCTA protocols, which caused a CT number shift at mCTA, the CT number bias value determined from the head phantom study was subtracted from the mCTA images. Of note, this bias correction is only required for our retrospective dataset, which was acquired with different collimator widths between NCCT and mCTA scans; prospectively, this correction can be avoided by acquiring NCCT and mCTA with the same collimator width.

To investigate mCTA-P studies free of potential CT number inconsistencies, we simulated an ideal mCTA-P-like study by down-sampling the standard CTP study to the four images corresponding to mCTA-P (down-sampled CTP, dCTP). The four images were selected by referring to the arterial TDC at CTP and comprised of (1) the pre-contrast baseline image (surrogate NCCT), the image at peak arterial enhancement (first phase of mCTA), and two delayed images at 8 s interval after the arterial peak (second and third phases of mCTA). This dynamic study simulated the case in which NCCT and mCTA were acquired with consistent scan parameters.

### Perfusion map processing

CTP, dCTP, and mCTA-P studies were processed independently using a prototype automated CTP software. Following motion correction by rigid in-plane registration to the baseline image of each study, dynamic images were linearly interpolated to a virtual sampling interval of 1 s. Arterial and venous TDCs were automatically detected by the software at a contralateral proximal middle cerebral artery or basilar artery and the sagittal sinus, respectively. Dynamic images were smoothed by a Gaussian filter with a full width half maximum of 4.8 mm. We used the Johnson-Wilson-Lee model^11^ to generate perfusion parametric maps. The model comprised the following parameters: time delay from contrast arrival at the artery to tissue (*T*_*0*_), minimum capillary transit time (*W*), perfusion (*F*), and the fraction (*E*) and clearance rate constant (*k*) of contrast with transit time greater than *W*. In this study, *k* was fixed to a value of 0, yielding a four-parameter model (*T*_*0*_, *W*, *F*, *E*). For CTP, *k* = 0 represents the slow capillary clearance for a small fraction of contrast, which was visible as residual contrast^12^ towards the end of the 60 s dynamic scan. For mCTA-P and dCTP, we used the same four-parameter model as we empirically found it to achieve an adequate balance of model simplicity and degrees of freedom to better estimate the perfusion parameters. Cerebral blood volume was computed as the area underneath the model impulse response function (Supplemental Fig. 1) and the mean transit time was the ratio of the blood volume and blood flow by the Central Volume Principle^13^. Tmax was defined as T_0_ + 0.5MTT.

Parametric maps of cerebral blood flow (CBF) and Tmax were generated by deconvolving the arterial TDC from each brain tissue TDC using a least-squares fitting algorithm. Lesion determination was limited to the ipsilateral brain hemisphere and hypoperfused tissue, which was defined as ipsilateral brain with Tmax > 4 s. The optimal ischemic core and penumbra thresholds for this software were CBF < 15% relative to the mean CBF in the contralateral brain hemisphere and Tmax > 6 s, respectively^14^. For dCTP and mCTA-P, the optimal ischemic core and penumbral thresholds were CBF < 25% and Tmax > 5 s, respectively, as determined by an independent threshold calibration experiment^14^ (“Supplemental Materials”). The disparity between CTP and mCTA-P lesion thresholds was due to the different CBF and Tmax estimations as a result of calculating perfusion maps with only four images (sparse sampling and shorter total scan duration compared to standard CTP; “Supplemental Materials”). Lesion segmentations were morphologically post-processed by closing, opening, and hole-filling operations, and disconnected segments less than 1 ml in volume were excluded from the final lesion segmentation.

### Statistical analysis

The primary outcome of this study was the agreement between CTP and mCTA-P in identifying a favourable/unfavourable mismatch profile (core volume < 70 ml, penumbra ≥ 15 ml, mismatch ratio ≥ 1.8)^2^. Agreement was quantified by the Cohen’s kappa coefficient (*κ*) and classification metrics such as sensitivity, specificity, and accuracy were also computed using the CTP determination as the reference. We interpreted *κ* < 0.00 as no agreement, 0.00–0.20 as slight, 0.21–0.40 as fair, 0.41–0.60 as moderate, 0.61–0.80 as substantial, and 0.81–1.00 as excellent agreement^15^. The secondary outcome was the agreement in lesion volume estimates between CTP and mCTA-P. Correlation between lesion volumes was assessed by the Pearson correlation coefficient (R) and volume agreement by Bland–Altman analysis. Bland–Altman differences were quantified as CTP minus the compared technique (dCTP or mCTA-P). Statistical analysis was performed on Prism (Version 9, GraphPad, San Diego, CA) using a two-tailed alpha of 0.05 for statistical significance.

## Results

### Patient characteristics

**Figure 1:**
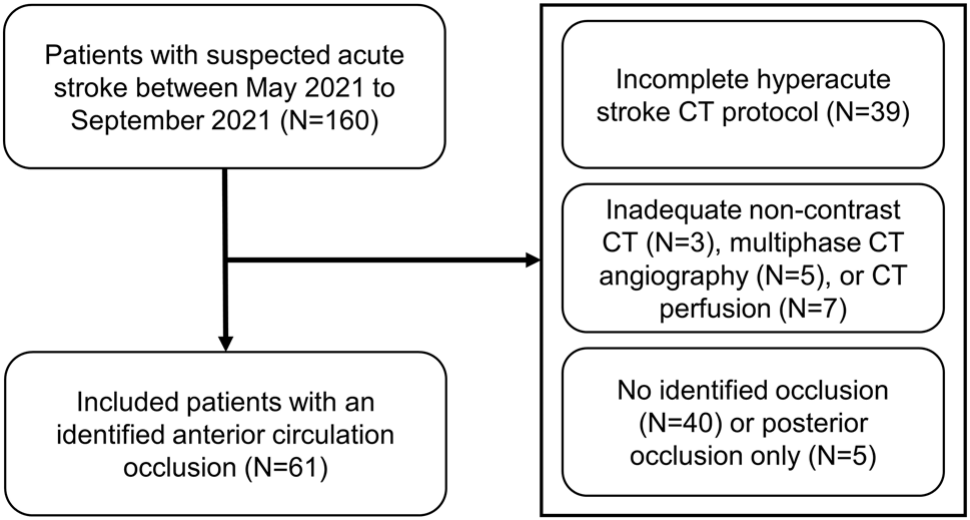
Flow chart of included patients in the study period.

Of 160 patients with a suspected acute stroke identified during the study period, 61 met the inclusion criteria. Figure 1 illustrates the inclusion/exclusion flowchart. Thirty-nine excluded patients did not have mCTA or CTP mainly due to presentation with an intracranial hemorrhage at NCCT or transfer from a primary stroke centre and comprehensive repeat imaging was deemed unnecessary. An additional 15 were excluded based on technically inadequate CT scans, mostly due to patient motion. Forty-five patients were excluded because an anterior circulation arterial occlusion was not detected. Patient demographics and clinical characteristics are summarized in Table 2.

**Table 2:** Patient demographics and clinical characteristics.

Parameter	Value
Age, years	75 (67–80)
Male (%)	31 (53)
Onset to CT, mins	242 (129–471)
CT to EVT completion time, mins*	81 (54–101)
NIHSS	9.0 (4.0–14.5)
Occlusion site, N (%)	
ICA	10 (14)
M1 MCA	27 (39)
M2 MCA	25 (36)
ACA/PCA/Distal MCA	8 (11)
Treatment, N (%)	
EVT + IV-tPA	12 (20)
EVT	15 (24)
IV-tPA	12 (20)
Medical management	22 (36)
mTICI, N (%)*	
0/1/2a	6 (22)
2b	3 (11)
2c	2 (7)
3	16 (60)
CT perfusion lesion volume	
Core [ml]	3.9 (0.0–19.1)
Penumbra [ml]	87.8 (30.3–145.4)
Down-sampled CT perfusion lesion volume	
Core [ml]	2.7 (0.0–16.3)
Penumbra [ml]	63.1 (25.0–129.4)
Multiphase CT angiography-perfusion lesion volume	
Core [ml]	1.1 (0.0–4.6)
Penumbra [ml]	56.5 (22.2–112.2)

Values are median (interquartile range) unless otherwise stated.

*EVT* indicates endovascular therapy; *IV-tPA* intravenous alteplase; *ICA* internal carotid artery; *MCA* middle cerebral artery; *ACA* anterior cerebral artery; *PCA* posterior cerebral artery; *mTICI* modified thrombolysis in cerebral infarction.

*in patients receiving EVT (N = 27).

### CT number consistency between NCCT and mCTA scan protocols

**Figure 2:**
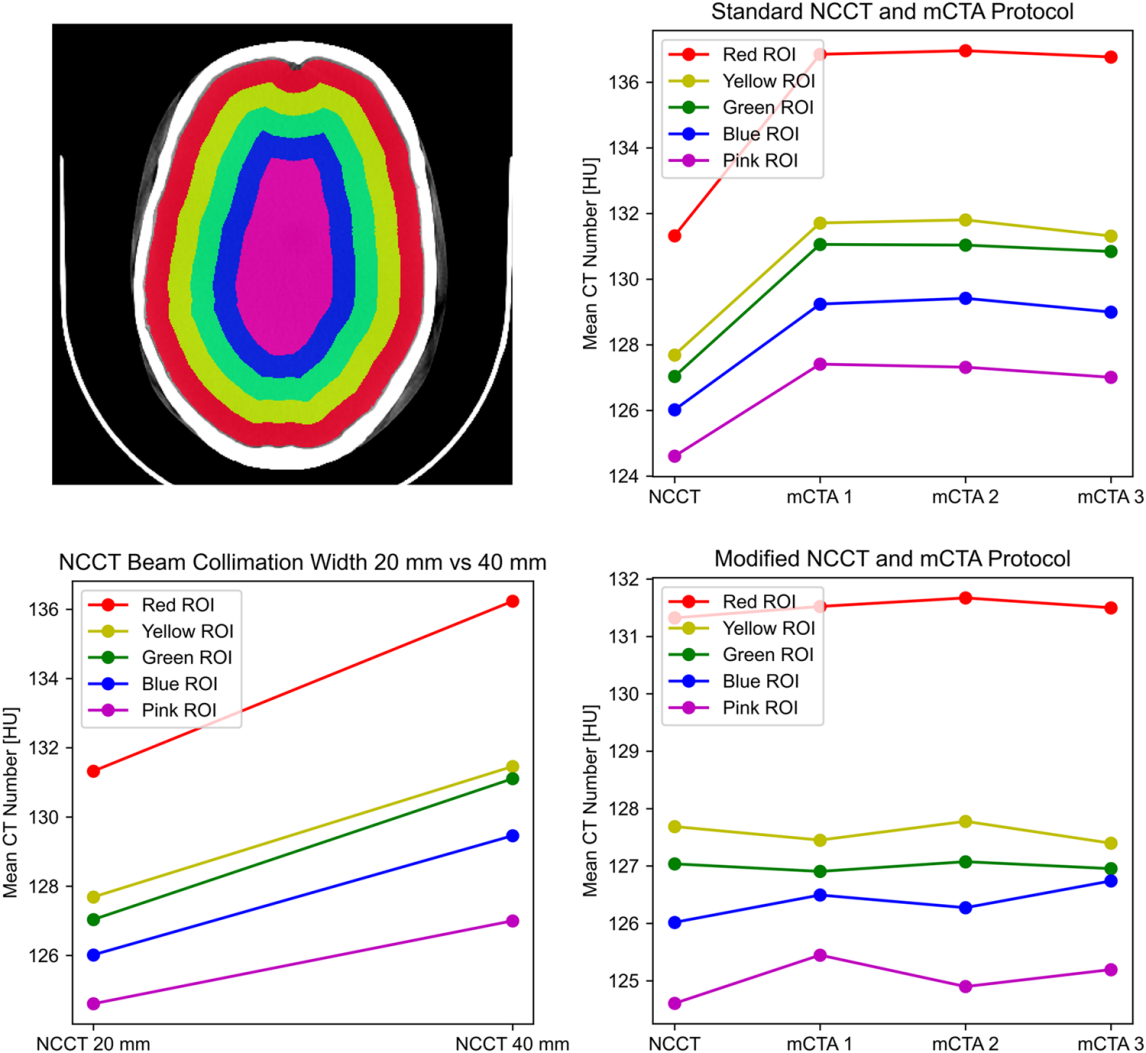
Region of interest (ROI) segmentations overlaid on a CT scan of an anthropomorphic head phantom (top left). In each plot, the line colour corresponds to the colour of the ROI in the segmentation. A substantial 3–5 HU increase in CT number is seen between the standard non-contrast CT (NCCT, 20 mm beam collimation width) and standard multiphase CT angiography (mCTA, 40 mm) protocols at all ROIs (top right). The effect of beam collimation width was isolated by scanning with the NCCT protocol at 20 mm and 40 mm beam collimation, which showed a similar increase in CT number at 40 mm (bottom left). A modified mCTA-perfusion protocol was tested in which mCTA beam collimation width was decreased to 20 mm to match that of NCCT, which substantially improved CT number consistency between NCCT and mCTA images (bottom right).

CT number results from phantom scans are summarized in Fig. 2. A clear increase in CT number by 3–5 HU was found with the standard mCTA scan protocol compared to that of the standard NCCT protocol. mCTA CT number overestimation was greater near the skull as opposed to more medial regions; that is, CT number inconsistency was spatially dependent. Increased CT number was also found when scanning with the standard NCCT protocol at 40 mm beam collimation width compared to 20 mm while keeping all other scan parameters unchanged. Furthermore, using a modified mCTA-P protocol, which changed the mCTA beam collimation width to 20 mm, better CT number consistency was achieved between NCCT and mCTA. Based on these findings, prior to calculating perfusion maps, a zeroth-order bias correction was applied by subtracting 4 HU in the brain voxels of real mCTA images in our dataset, which were acquired with the standard mCTA scan parameters. The effect of CT number bias correction on calculated mCTA-P maps is illustrated in Supplemental Fig. 3. An example of model fitted TDCs for CTP, dCTP, and mCTA-P are shown in Supplemental Fig. 4.

### Perfusion-based selection criteria

**Figure 3:**
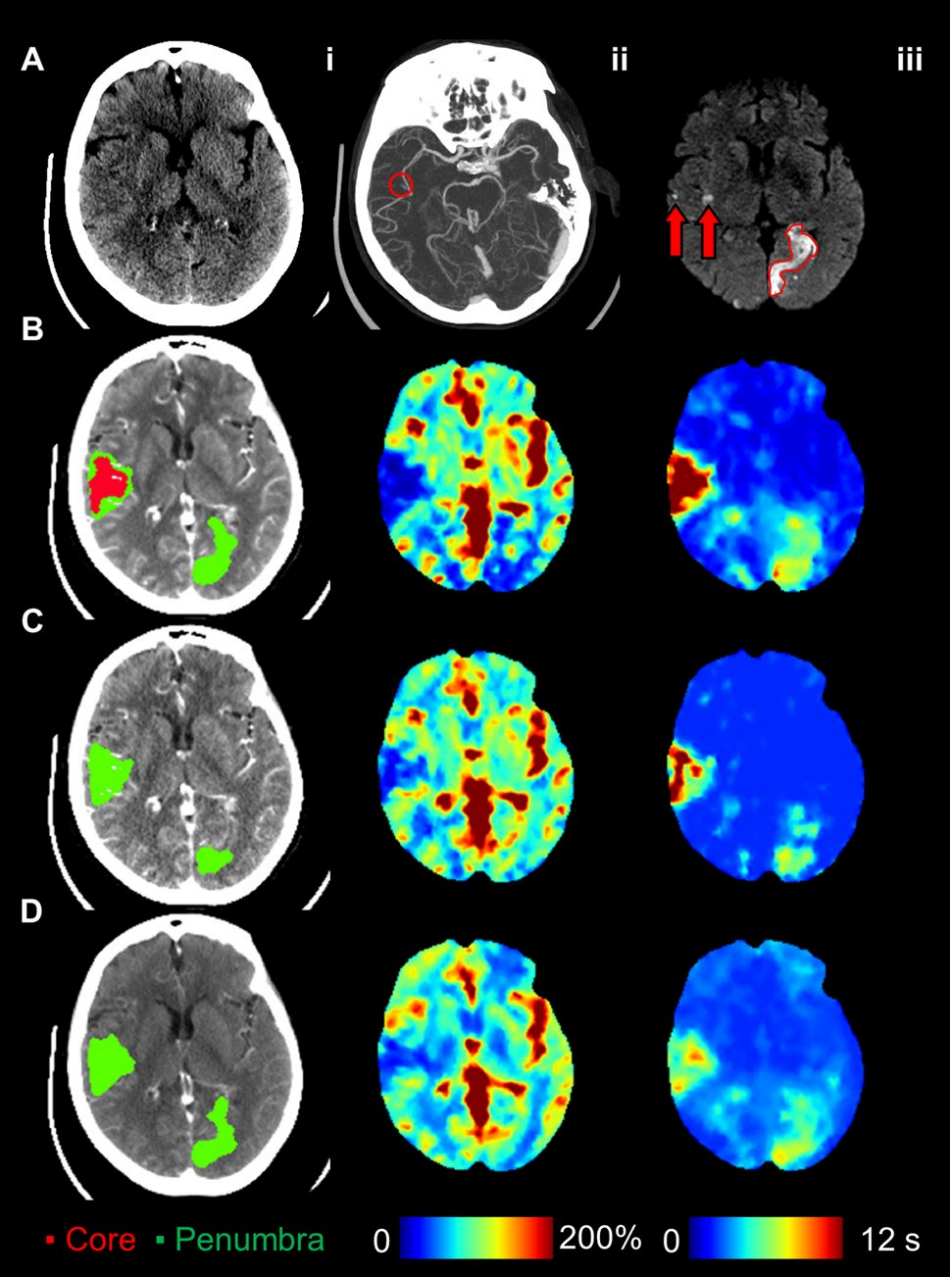
Patient presenting with unknown time from stroke symptom onset, a bilateral occlusion of the right M2 middle cerebral artery (MCA) and left distal posterior cerebral artery (PCA), and an NIHSS of 10. This patient received endovascular thrombectomy for the right M2 occlusion within 53 min of admission CT (mTICI = 3), but the posterior occlusion could not be treated. (**A**.**i**) Admission non-contrast CT; (**A**.**ii**) maximum intensity projections of CT angiography with the red circle indicating the occlusion site; (**A**.**iii**) follow-up diffusion-weighted imaging showing regions of infarction in the right M2 territory (red arrows) and the left posterior brain (red outline). (**B**.**i**) CT perfusion ischemic core (red, 19.4 ml) and penumbra (green, 45.5 ml; mismatch ratio of 2.3) derived from cerebral blood flow (**B**.**ii**) and Tmax maps (**B**.**iii**) indicated a favourable DEFUSE-3 mismatch profile. (**C**) Similarly, down-sampled CT perfusion (dCTP) indicated a favourable mismatch profile with an ischemic core and penumbral volumes of 4.8 ml and 26.9 ml, respectively (mismatch ratio 5.6). (**D**) Multiphase CT angiography-perfusion (mCTA-P) agreed with the favourable CTP mismatch profile with ischemic core and penumbral volumes of 1.2 ml and 51.2 ml, respectively (mismatch ratio 44.3). Of note, the indicated lesion volumes were only of the right MCA territory so that the left PCA lesion volumes did not affect the estimation of mismatch.

**Figure 4:**
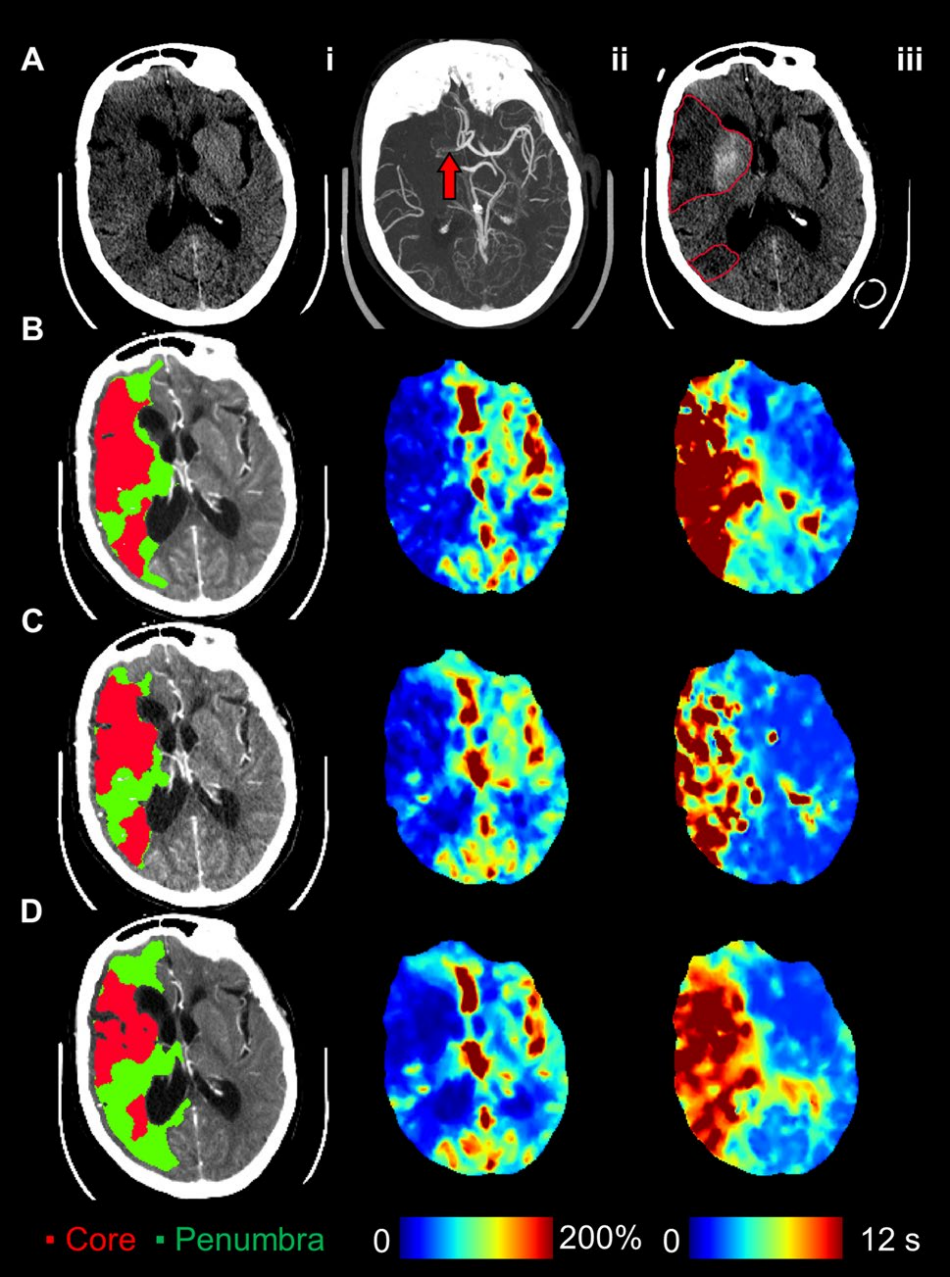
Patient presenting 5 h after stroke symptom onset with an occlusion of the right internal carotid to M1 middle cerebral artery and an NIHSS of 22. This patient did not receive reperfusion treatment. (**A**.**i**) Admission non-contrast CT shows early ischemic changes in the right MCA territory; (**A**.**ii**) maximum intensity projections of CT angiography with the red arrow indicating the occlusion site; (**A**.**iii**) follow-up non-contrast CT showing infarction and hemorrhage outlined in red. (**B**.**i**) CT perfusion ischemic core (red, 84.0 ml) and penumbra (green, 236.8 ml; mismatch ratio of 2.8) derived from cerebral blood flow (**B**.**ii**) and Tmax maps (**B**.**iii**). DEFUSE-3 mismatch was unfavourable by CT perfusion due to the large core volume. (**C**) Similarly, down-sampled CT perfusion (dCTP) indicated an unfavourable mismatch profile with an ischemic core and penumbral volumes of 71.7 ml and 205.3 ml, respectively (mismatch ratio 2.9). (**D**) Multiphase CT angiography-perfusion (mCTA-P) showed a similar ischemic lesion, but underestimated ischemic core and penumbra at 58.0 ml and 202.5 ml, respectively (mismatch ratio 3.5), leading to a favourable DEFUSE-3 mismatch profile and disagreement with CTP.

Table 3 summarizes the agreement and accuracy of classifying CTP diagnostic criteria with dCTP and mCTA-P. Forty-six patients had a favourable mismatch profile as determined by standard CTP. A substantial agreement (*κ* = 0.75, 95% confidence interval [CI]: 0.56–0.94) was found between CTP and dCTP mismatch profiles whereas agreement was moderate with mCTA-P (*κ* = 0.48, 95% CI: 0.22–0.74). Using CTP mismatch profiles as the ground truth, classification accuracy was 90% with dCTP and 82% with mCTA-P. Overall, mCTA-P had weaker specificity for identifying an unfavourable mismatch than sensitivity for a favourable profile. Figures 3 and 4 show examples of a concordant and discordant mismatch profile using mCTA-P versus CTP.

**Table 3:** Agreement (*κ*), sensitivity, specificity, and accuracy of target mismatch profile identified by down-sampled CT perfusion and multiphase CT angiography-perfusion versus standard CT perfusion.

	*κ* (95% CI)	Sensitivity (%)	Specificity (%)	Accuracy (%)
Down-sampled CT perfusion	0.75 (0.56–0.94)	42/46 (91)	13/15 (87)	55/61 (90)
Multiphase CT angiography-perfusion	0.48 (0.22–0.74)	42/46 (91)	8/15 (53)	50/61 (82)

*κ* is the Cohen’s kappa coefficient; CI indicates confidence interval.

Of the 11 patients with misclassified mismatch profiles by mCTA-P, median age was 77 (interquartile range, IQR: 67–85) years, 6 (54%) were female, median time from stroke onset to CT was 313.5 (IQR: 192.5–403.5) mins, median NIHSS was 3.5 (IQR: 2–11), 7 of 11 (64%) had an M2 occlusion, 3 (27%) received IV-tPA, 1 (9%) underwent EVT (modified thrombolysis in cerebral infarction, mTICI = 0)^16^, and the remaining patients (64%) received medical management. For the 6 patients with misclassified mismatch profiles by dCTP, median age was 90 (IQR: 77–92.5) years, 2 (33%) were female, median time from stroke onset to CT was 546.0 (289.5–795.0) mins, median NIHSS was 9 (IQR: 2–13), 3 (50%) had an M2 occlusion, 1 (17%) underwent EVT (mTICI = 2c), and the remaining patients (83%) received medical management. Two patients had misclassified mismatch profiles by both dCTP and mCTA-P.

### Ischemic lesion volume accuracy

**Figure 5:**
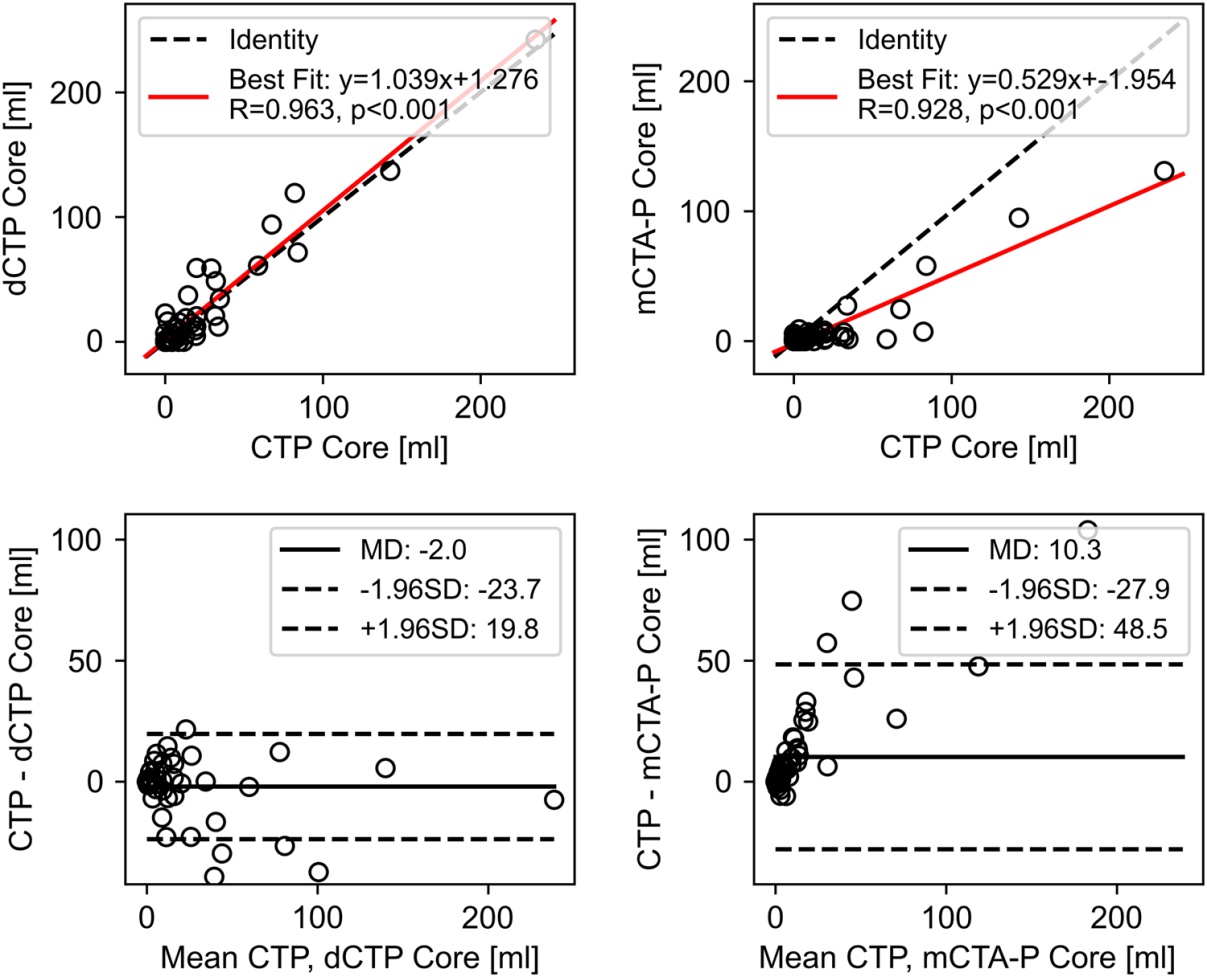
Correlation (top row) and Bland–Altman plots (bottom row) of CT perfusion (CTP) versus (left) down-sampled CTP (dCTP) and (right) multiphase CT angiography-perfusion (mCTA-P) ischemic core volume. R indicates the Pearson correlation coefficient; MD, mean difference; ± 1.96SD, standard deviation, indicate the 95% limits of agreement.

**Figure 6:**
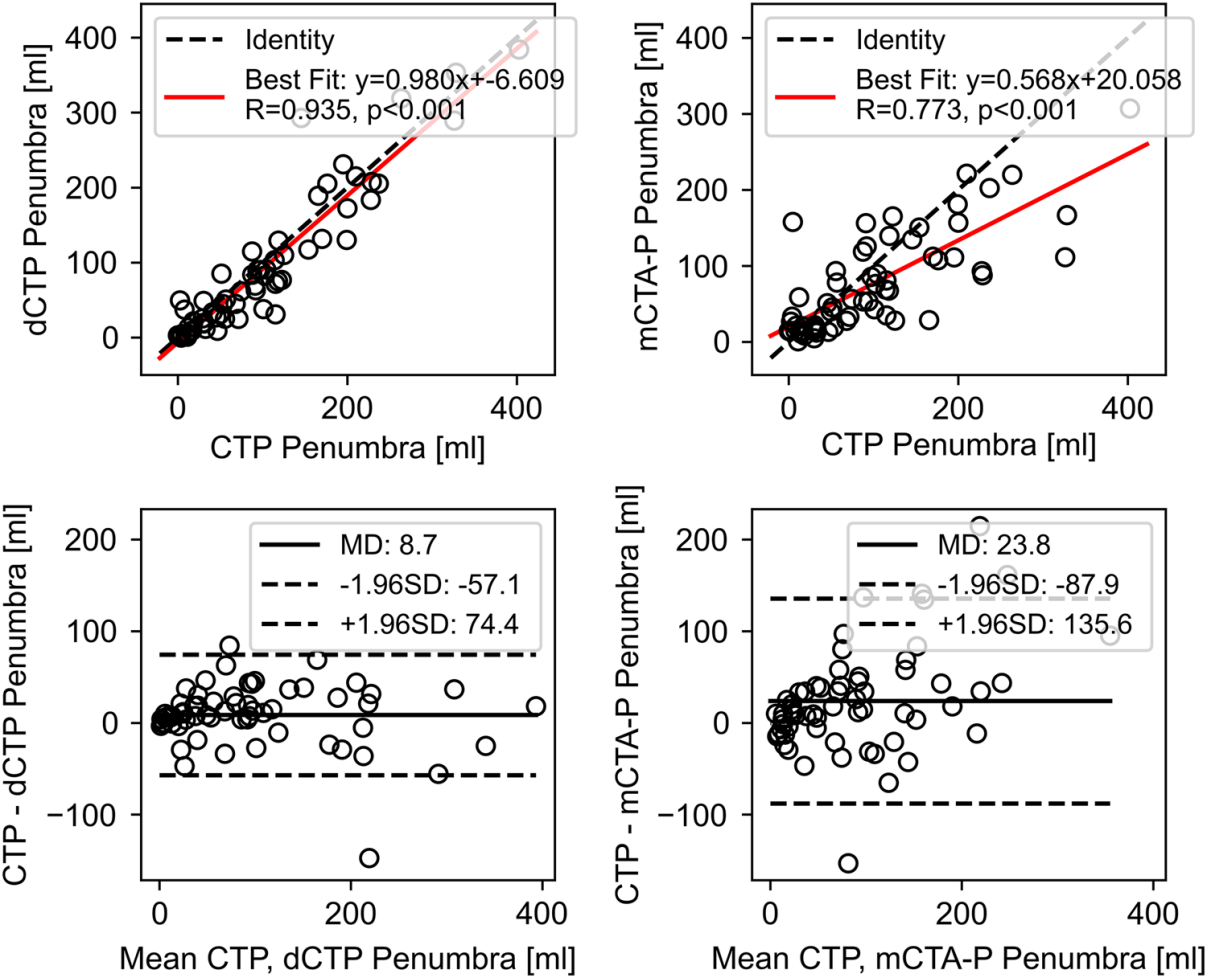
Correlation (top row) and Bland–Altman plots (bottom row) of CT perfusion (CTP) versus (left) down-sampled CTP (dCTP) and (right) multiphase CT angiography-perfusion (mCTA-P) ischemic penumbra volume. R indicates the Pearson correlation coefficient; MD, mean difference; ± 1.96SD, standard deviation, indicate the 95% limits of agreement.

Correlation and Bland–Altman plots comparing CTP lesion volumes against those from dCTP and mCTA-P are shown in Figs. 5 and 6. Down-sampled CTP and mCTA-P ischemic core volumes had strong correlation and agreement to that of CTP, albeit with underestimation by mCTA-P. Penumbral volume correlation and agreement to CTP remained strong with dCTP but was substantially underestimated with mCTA-P and had weaker correlation. Overall, all CTP lesion volumes were underestimated by mCTA-P, CTP core and penumbra volumes were marginally overestimated and underestimated with dCTP, respectively.

## Discussion

In this study, we explored the feasibility of calculating surrogate perfusion maps from a shorter stroke CT protocol that requires less expertise, time, and radiation exposure. Multiphase CTA is a simple extension to single-phase CTA and can be easily incorporated at any EVT-screening hospital. We compared perfusion mismatch and ischemic lesion volumes predicted from a perfusion imaging sequence comprised of NCCT and mCTA against a CTP reference. Our study demonstrated moderate agreement between CTP and mCTA-P perfusion mismatch criteria and ischemic lesion volumes, but this may be improved with better optimized scan protocols (i.e., demonstrated by dCTP). Of note, dCTP represented mCTA-P acquired with equal collimator width between NCCT and mCTA, which achieved substantial agreement in mismatch profiles with CTP. Ischemic core volumes determined by dCTP and mCTA-P had strong correlation and agreement to those from CTP, but mCTA-P lesion volumes substantially underestimated those from CTP. Taken together, mCTA-P may be a useful alternative to CTP in environments where a reliable CTP study cannot be acquired or when the risks associated with additional radiation dose, contrast volume, and possible delays to patient management outweigh the potential benefits of a separate CTP study. It may prove helpful with patient motion, which often worsens with time lying on the CT table and results in technically inadequate CTP when acquired after NCCT and mCTA.

Our study had two main contributions. First, mismatch profiles could be determined from perfusion maps generated from only four dynamic images (i.e., mCTA-P, which is a low temporal resolution and short-duration CTP study). Prior work has suggested that scan interval between dynamic CTP images should be no more than 3–5 s^17,18^. In contrary, our study demonstrated that sampling intervals can be as long as 8 s as in mCTA to determine perfusion mismatch profiles. Our approach differs from previous studies in that (1) mCTA is timed to sample the peak enhancement of the brain arteries, whereas prior studies uniformly subsampled dynamic images to increase scan interval, so peak enhancement may have been missed; (2) this software used a model-based deconvolution method, which is more robust to low temporal resolution and short-duration scans compared to model-independent methods such as singular value decomposition^18,19^.

Second, perfusion map quality and mismatch profile assessment are greatly affected by scan parameter selection, so NCCT and mCTA protocols must be calibrated to maintain CT number consistency between dynamic images. The importance is highlighted by the substantial difference in diagnostic reliability between dCTP and mCTA-P. This is a simple adjustment to NCCT and mCTA scan parameters such that they use the same kV (to maintain consistent iodine sensitivity) and beam collimation width (to maintain consistent levels of X-ray scatter). For clinical evaluation of mCTA for occlusion detection and assessment of collaterals, a small CT number shift as we observed (Fig. 2) may not be relevant compared to > 500 HU intravascular enhancement. However, the effect of this CT number shift is proportionally greater for TDCs, which may only have an amplitude of 10–15 HU in normal grey matter. This fixed bias disproportionately affects TDCs with lower CBF and cerebral blood volume (i.e., ischemic tissue) because their peak enhancement is smaller (e.g., 2–5 HU). As such, CBF contrast decreased in mCTA-P maps (Supplemental Fig. 3), resulting in an underestimation of stroke lesion volumes as we have observed in our study. The NCCT and mCTA images used in this study were from a retrospective clinical database, so beam collimation width was not tuned specifically for ensuring the CT number consistency required in mCTA-P. This finding may also have implications for other protocols that combine CTA and CTP imaging (i.e., dynamic or 4D-CTA)^20^. In whole-brain CT scanners with up to 16-cm total collimation width, X-ray scatter levels may be greatly different between collimator width settings and thus scan parameter selection is especially important.

While a reliable CTP acquisition may still be preferred, mCTA-P may be a suitable alternative when CTP cannot be reliably acquired due to unavailable technical expertise or is not strictly indicated by guidelines (e.g., within 6 h of stroke onset) or in consideration of radiation dose, contrast injections, patient compliance, or logistic obstacles resulting in delays to appropriate patient management. In Ontario, Canada, NCCT and mCTA are universally acquired at all hospitals receiving stroke patients, but CTP is mostly only available at urban centres^9^. Availability of CTA and CTP may vary between health care systems and countries, but it is generally expected that CTA is more routine and prevalent than CTP^7^. With our proposed mCTA-P technique, automated assessment of ischemic lesion volumes and perfusion-mismatch criteria may therefore be made more readily available at centres without the capacity for routine CTP.

CTP remains controversial in the acute stroke imaging community as the diagnostic value of mismatch-based selection remains uncertain. Recent large core EVT trials suggested that patients with traditionally unfavourable mismatch profiles may still benefit from late-window EVT compared to medical therapy^21–23^. Non-randomized cohort studies also suggested that NCCT alone (with large vessel occlusion detected at CTA) may be sufficient for selecting patients for late-window EVT, potentially obviating the need for CTP^24,25^. In contrast, Sarraj et al. reported that a favourable versus unfavourable CTP profile was more strongly associated with good versus poor functional outcome compared to a favourable versus unfavourable NCCT profile by the Alberta Stroke Program Early CT Score (ASPECTS)^26^. The inter-rater reliability of NCCT-ASPECTS may be limited^27,28^, so our proposed tool may augment readings of NCCT-ASPECTS with an estimation of ischemic core. A recent single-centre study also suggested that unfavourable mismatch profiles and CTP parameters were independently associated with poor treatment outcomes (e.g., symptomatic intracranial hemorrhage and death) in the early EVT time window^29^. Additionally, perfusion imaging may still add diagnostic value^30^ by quantifying ischemic lesion volumes, guiding the detection of intracranial occlusions^31–36^, and potentially ruling out unfavourable NCCT profiles (i.e., low ASPECTS)^26^. With these conflicting pros and cons, a separate CTP study may not be desirable in the early time window, whereas mCTA-P has the potential to provide similar diagnostic information using the ubiquitous NCCT and more widely available mCTA, without introducing additional radiation dose, contrast injections, or imaging time. Additionally, mCTA-P can provide whole brain coverage with helical scanning enabled by the long scan intervals, though this benefit was not explored to facilitate consistent analysis with CTP (8 cm brain coverage). Reader studies evaluating the potential benefit of mCTA-P maps in detecting ischemia and occlusions will be the subject of a future study.

The optimal ischemic core threshold for our software was CBF < 15% for CTP^14^ and CBF < 25% for mCTA-P (“Supplemental Materials”) and not CBF < 30% as with RAPID CTP (RapidAI, Menlo Park, CA) used in clinical trials. Optimal stroke lesion thresholds vary by CTP software^14,37,38^ and appropriate thresholds must accordingly be applied. We also found that stroke lesion thresholds for mCTA-P was different to those of CTP due to the 4-image protocol (“Supplemental Materials”), which used substantially fewer images compared to a standard CTP protocol.

This study had limitations. First, this was a retrospective study of previously acquired NCCT and mCTA scans of stroke patients. Because mCTA scans had greater scatter from a wider collimator width used, scatter correction determined with a phantom had to be applied. In prospective studies, the collimator width in both NCCT and mCTA scans can be set to be the same, obviating the need for scatter correction, and would lead to improved perfusion map quality. CT number inconsistency caused by greater scatter at mCTA also resulted in poorer normal-to-ischemic contrast in the mCTA-P CBF and Tmax maps (Supplemental Fig. 3), resulting in underestimated ischemic lesion volumes and poorer mismatch profile specificity. Based on the dCTP results, which simulated an mCTA-P study free of CT number inconsistency, we expect that when using consistent scan protocols between NCCT and mCTA, ischemic lesion volume accuracy and mismatch profile specificity will improve. As well, data were from a single tertiary centre using a single CT scanner. However, we expect that if consistent beam collimation width and tube voltage are used in both NCCT and mCTA, then further corrections to standard reconstructions will not be required to generate diagnostic quality perfusion maps. Second, due to the retrospective inclusion of patients managed by standard of care, clinical endpoints such as 90-day functional outcome were not available to determine associations between imaging markers and outcome. Third, although dCTP was meant to simulate an ideal mCTA-P acquisition, there were differences in their scan parameters such as tube voltage (80 kV vs 120 kV), tube current-exposure time product (76 mAs vs 250 mAs at NCCT and ~ 78 mAs at mCTA), axial versus helical scanning, and injected contrast volume (40 ml at CTP vs 80 ml at mCTA). Importantly, because the tube voltage between NCCT and mCTA and between dynamic CTP images were consistent, we expect TDCs to vary linearly with iodine concentration across the mCTA-P and dCTP studies. As well, despite mCTA using double the contrast volume, we do not expect a great difference in SNR between dCTP and mCTA-P because a higher tube voltage of 120 kV was used, leading to decreased iodine sensitivity compared to at 80 kV for CTP. The accuracy of mCTA arterial phase timing also requires further investigation, though we expect relatively low timing error in our study due to our use of a test bolus method for CTA scan timing. Contrast injection protocols and mCTA scan timing are nonetheless two scan parameters deserving of further characterization.

Additionally, there was a chance of selection bias as this was a retrospective study of patients specifically with anterior circulation ischemic stroke. A substantial number of patients were excluded from the study due to incomplete imaging, no identified occlusion, or inadequate scans. Many patients were excluded from the study primarily because of our inclusive approach to identifying potential participants. Specifically, we considered any patient presenting with a suspected stroke during the four-month study period. Furthermore, we mitigated the risk of selection bias by including consecutive patients. Only patients with anterior circulation ischemic strokes were included to control for the effect of occlusion site and because it is the main target for EVT^39^. The role of perfusion imaging and our proposed technique for identifying posterior stroke treatment targets as well as stroke mimics require clarification in future studies. Lastly, the low number of late presenters limited the assessment of our technique in patients for whom perfusion imaging is mainly indicated. At our institution, multimodal CT including CTP is standard of care for all patients presenting with a suspected stroke, so analysis of this patient cohort is still relevant to centres like ours. As well, perfusion-mismatch is still valid in the early time window although not strictly required to identify patients for treatment^3,26,29,30^. A prospective comparison of mCTA-P and CTP to select patients for EVT is required to validate our proposed technique in clinical practice.

Perfusion mismatch criteria for CT-based acute ischemic stroke triage is currently only available via advanced CTP imaging. In this study, we demonstrated the feasibility of determining mismatch profiles and ischemic lesion volumes with standard NCCT and mCTA imaging. We highlight the need for controlling scatter by using consistent beam collimation widths such that CT number is consistent between NCCT and mCTA. Proper implementation of NCCT and mCTA-derived perfusion may allow reliable perfusion mismatch assessment when CTP cannot be acquired or the risks of additional radiation dose, contrast injections, and imaging time outweigh the potential benefit of a separate CTP study.

### Supplementary Information

Supplementary Information.

## Data Availability

The dataset used in this study may be available upon reasonable request to the corresponding author.
