# Supplementary material for: Multiphase CT angiography perfusion maps for predicting target mismatch and ischemic lesion volumes
Source: Sci Rep. 2023 Dec 11;13:21976. doi: 10.1038/s41598-023-48832-9 (PMC10713587; doi:10.1038/s41598-023-48832-9)
Supplement: Supplementary file 1 — Supplementary Information. [file 41598_2023_48832_MOESM1_ESM.pdf]

Supplemental Materials for "Multiphase CT Angiography Perfusion Maps for Predicting Target Mismatch and Ischemic Lesion Volumes"

Kevin J. Chung,[1,2] Sachin K. Pandey,[3] Alexander V. Khaw,[4] Ting-Yim Lee[1-3*]

[1]Department of Medical Biophysics, The University of Western Ontario, London, ON

[2]Robarts Research Institute and Lawson Health Research Institute, London, ON

[3]Department of Medical Imaging, The University of Western Ontario, London, ON

[4]Department of Clinical Neurological Sciences, The University of Western Ontario, London, ON

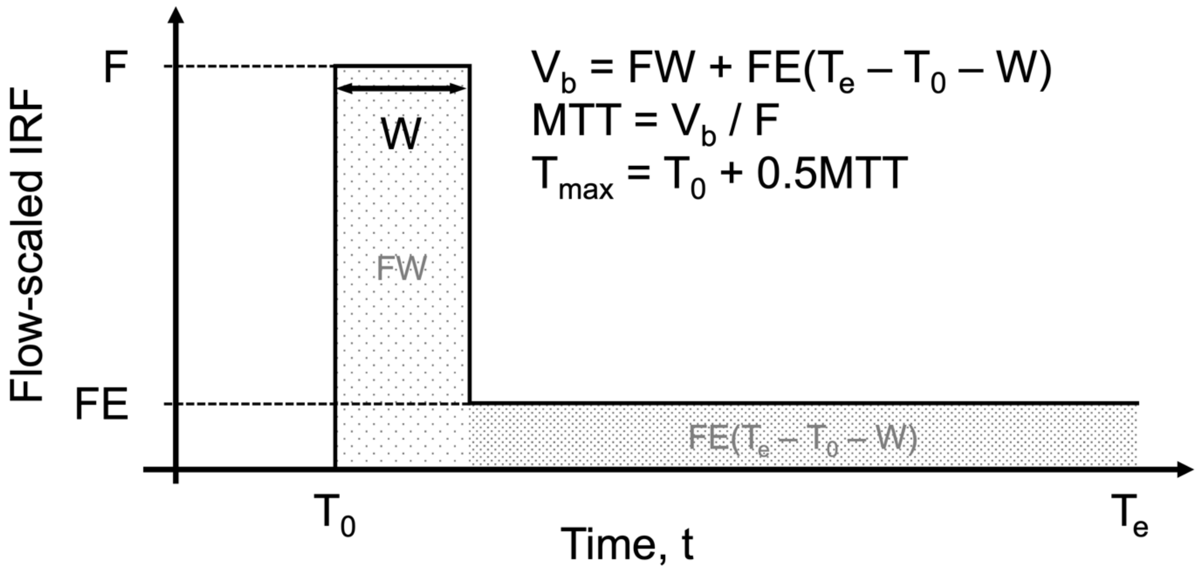

Supplemental Figure 1. The flow-scaled impulse residue function (IRF) of the Johnson-Wilson-Lee model with $k = 0$. The model has four free parameters (T_0, W, F, E): time delay from contrast arrival at the artery to tissue (T_0), minimum capillary transit time (W), blood flow (F), and the fraction (E) of contrast with transit time greater than W. The blood volume (V_b) was computed as the area underneath the flow-scaled IRF until the scan end time T_e and the mean transit time (MTT) was the ratio of the blood volume to blood flow by the Central Volume Principle.[1] For this model, Tmax was defined as T_0 + 0.5MTT.

Calibrating Ischemic Core and Penumbra Thresholds for Multiphase CTA-Perfusion

In standard CT perfusion (CTP) protocols, tissue time-density curves (TDC) are uniformly sampled at relatively short intervals (2 to 3 s per dynamic image). Our proposed non-contrast CT (NCCT) and multiphase CT angiography (mCTA) dynamic series (multiphase CTA-perfusion, mCTA-P) instead samples four images at relatively long sampling intervals: (1) baseline image prior to when contrast enters the brain (NCCT), (2) image at the peak enhancement of the brain arteries (first phase of mCTA), and (3, 4) two delayed images at 8 s intervals (second and third phases of mCTA). Due to the substantial differences in TDC sampling patterns, mCTA-P may have different biases in estimating cerebral blood flow (CBF) and Tmax, which in turn requires ischemic core and penumbra thresholds to be adjusted.

Ischemic core and penumbra thresholds for mCTA-P were calibrated to match that of standard CTP thresholds using a digital perfusion phantom-based technique. Briefly, the idea is to establish the quantitative accuracy by linear regression of estimated CBF and Tmax with each scan protocol (CTP and mCTA-P) against ground truth perfusion in a digital perfusion phantom. Then, linear regressions are compared between scan protocols to determine a calibration relationship. The method is briefly described below.

A digital perfusion phantom[2] comprises simulated tissue TDCs, which are generated by convolving an assumed arterial TDC and simulated IRFs with known ground truth perfusion parameters. The arterial TDC was taken from the internal carotid artery in the healthy brain hemisphere of a CTP study acquired at a uniform image interval of 1.8 s over 81 s. Flow-scaled IRFs were simulated as gamma-variate functions with a wide range of ground truth perfusion parameters: $T_0 \in [0.0, 0.5, 1.0, 2.0, 3.0, 4.0, 8.0]$ s, $MTT \in [3.4, 4.0, 6.0, 8.0, 10.0, 12.0, 16.0]$ s, and $CBV \in [0.5, 1.0, 1.5, 2.0, 2.5, 3.0, 4.0, 5.0]$ ml/100 g. CBF was calculated as CBV/MTT by the Central Volume Principle;[1] accordingly, CBF ranged from 1.9 to 88.2 ml/min/100 g at non-

uniform intervals. Ground truth tissue TDCs were calculated by numerically convolving the simulated IRF and the linearly interpolated patient arterial curve at 0.01 s interval then resampled at 2 s interval. Zero-mean Gaussian noise with standard deviation, $\sigma = 1.5$ HU was added to the tissue TDCs to simulate the expected noise variation in tissue TDCs after Gaussian filtering of dynamic CTP images at a strength of 4.8 mm (full-width half maximum) as used in this study. In total, 1024 noisy tissue TDCs were generated for each set of perfusion parameters by random sampling of Gaussian distributions with σ=1.5 HU.

The complete digital perfusion phantom was used to represent a standard CTP protocol (2 s interval over 80 s) and the mCTA-P protocol was simulated by down-sampling the dynamic images of the digital perfusion phantom to (1) the pre-contrast baseline image, (2) arterial peak image, and (3, 4) images at 8 and 16 s after the arterial peak. We refer to the full digital perfusion phantom as the CTP phantom and the down-sampled one as the dCTP phantom. CBF and Tmax were calculated by deconvolution of the arterial TDC from each tissue TDCs in the CTP phantom and the dCTP phantom.

Mean estimated CBF and Tmax were computed over the 1024 TDCs simulated for each set of perfusion parameters. These means were plotted against ground truth CBF and Tmax in the digital perfusion phantom and linear regression lines were calculated. Taking CTP CBF<15% and Tmax>6 s as the reference thresholds for ischemic core and penumbra, respectively,[3] the calibrated thresholds for the dCTP protocol were computed by comparing the linear regression lines.

Supplemental Figure 2 shows linear regression between ground truth CBF and Tmax in the digital perfusion phantom versus estimated CBF and Tmax for the CTP phantom and the dCTP phantom. Taking a ground truth CBF of 50 ml/min/100 g as normal CBF in the

contralateral hemisphere (for normalization to relative CBF), the expected normal CBF at the standard CTP and dCTP protocols were 47.9 and 49.9 ml/min/100 g, respectively. The reference CTP CBF<15% threshold was then 7.2 ml/min/100 g, which was equivalent to a ground truth CBF of 3.2 ml/min/100 g by the CTP regression line. Substituting this value into the regression line of the dCTP phantom, the equivalent absolute CBF 12.0 ml/min/100 g and thus a relative CBF of 24%. Similarly, using CTP Tmax>6 s as the reference penumbral threshold, the calibrated dCTP Tmax threshold was 4.8 s. For application on patient studies, the ischemic core and penumbral thresholds were rounded to CBF<25% and Tmax>5 s.

This method was used to systematically determine the optimal dCTP/mCTA-P threshold for ischemic core and penumbra based on system performance characteristics, rather than empirically determining the threshold that best achieves volume agreement to CTP. However, this phantom-based technique is a linear calibration method that may not fully account for non-linear relationships between the reference and the calibrated method. An example of a non-linear effect may be that caused by different levels of x-ray scatter between scans, thus resulting in CT number inconsistency between dynamic images. CT number inconsistency was found using our NCCT and mCTA protocols, which likely nonlinearly affected the estimated CBF and Tmax. As such, these calibrated thresholds are better suited for the dCTP series that we investigated, in which CT number inconsistency was not a problem. Better volume agreement to CTP may be achieved by further adjusting mCTA-P thresholds, but this was outside the scope of our study.

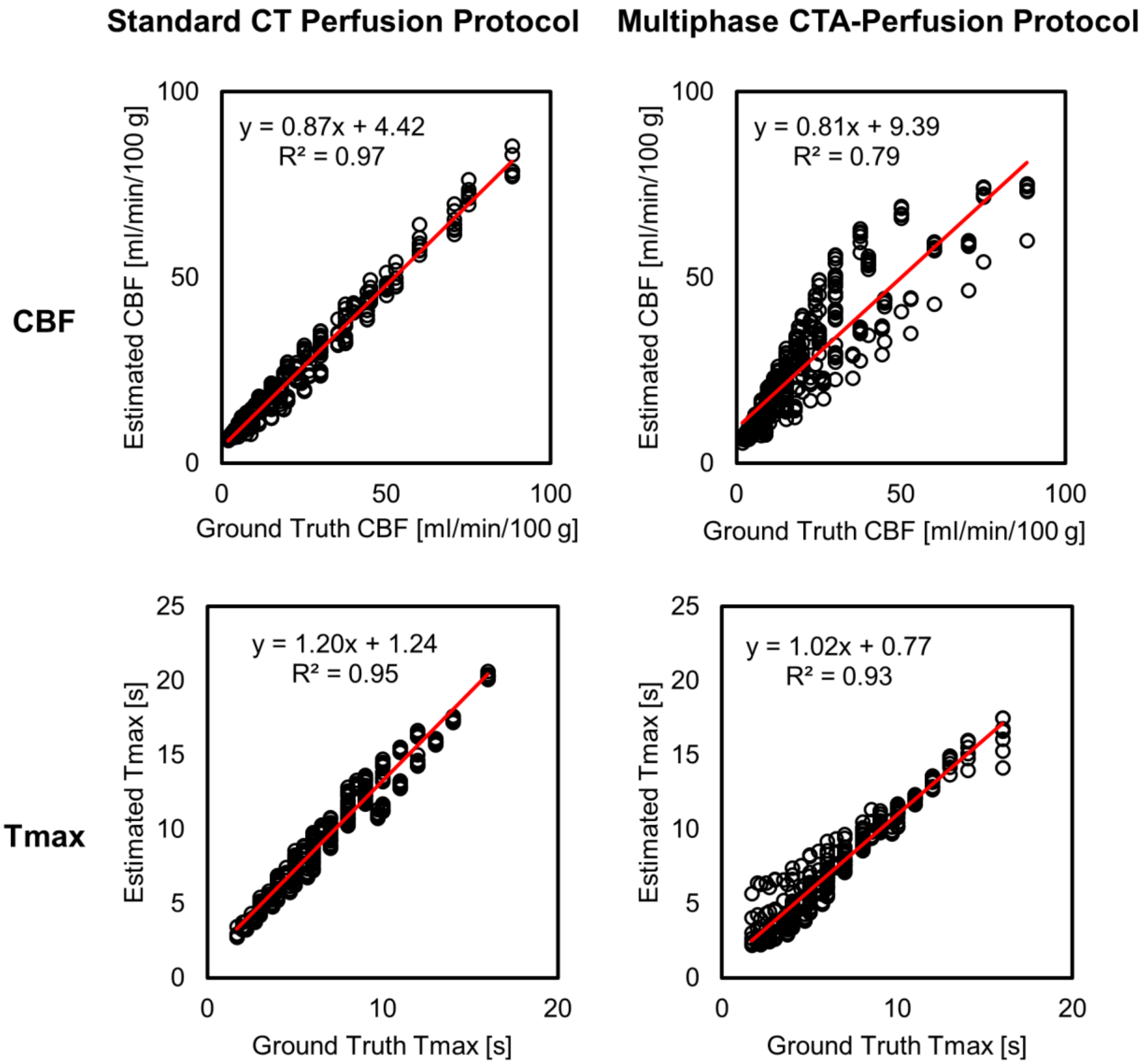

Supplemental Figure 2. Linear regression of ground truth cerebral blood flow (CBF; top row) and Tmax (bottom row) versus estimated CBF and Tmax using the standard CT perfusion protocol (left column) and the multiphase CT angiography (CTA)-perfusion protocol. The red lines are the linear best fit to the plotted data and their equations and goodness of fits (R^2) are indicated in each plot. As indicated by the slopes, intercepts, and goodness of fits, there are differences in estimated CBF and Tmax between the two protocols, necessitating an adjustment of ischemic core and penumbra thresholds at the multiphase CTA-perfusion protocol.

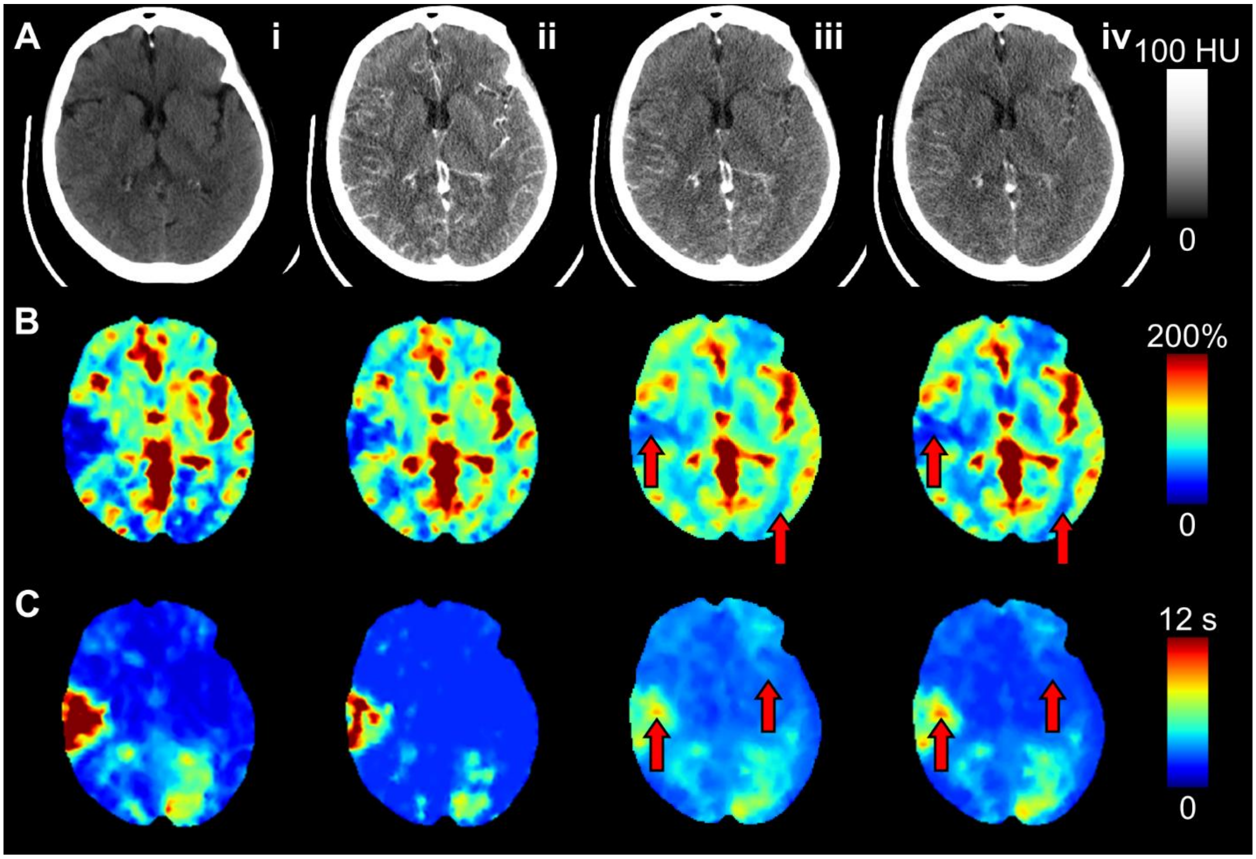

Supplemental Figure 3. Effect of CT number bias correction on multiphase CT angiography (mCTA)-perfusion maps for patient presented in Figure 2. (A) The mCTA-perfusion series comprised of (i) non-contrast CT and (ii to iv) mCTA phases 1 to 3. (B) Relative cerebral blood flow (CBF) and (C) Tmax maps of the same slice shown in (A) for (i) standard CT perfusion, (ii) down-sampled CT perfusion, (iii) mCTA-P without and (iv) with bias correction by 4 HU on mCTA images. After bias correction, there was an improvement in contrast between normal and ischemic mCTA-P CBF (red arrows, B.iii-iv), which better matched CT perfusion (B.i). Similarly, normal versus ischemic mCTA-P Tmax improved after bias correction (red arrows, C.iii-iv), which better matched CT perfusion (C.i).

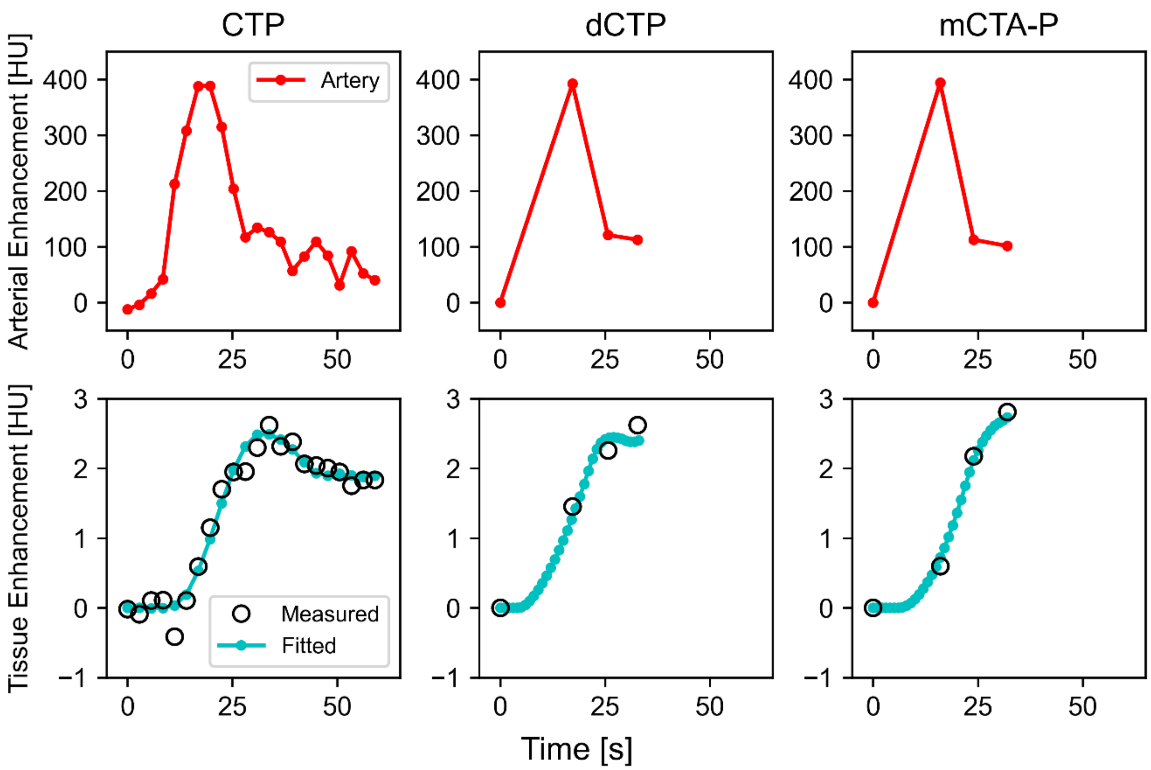

Supplemental Figure 4. Arterial (top row) and tissue (bottom row) time-density curves (TDC) overlaid with the model fitted tissue curve (teal line) for CT perfusion (CTP; left), down-sampled CTP (dCTP; middle), and multiphase CT angiography-perfusion (mCTA-P; right). The tissue region of interest for all three dynamic series was the ischemic core region of the patient in Figure 4 as identified by standard CTP (CBF<15% and Tmax > 6 s). Bias correction was applied on mCTA-P. Estimated model parameters are listed in the Supplemental Table.

Supplemental Table. Estimated model parameters for Supplemental Figure 4

Parameter	CTP	dCTP	mCTA-P
CBF [ml/min/100 g]	2.8	5.6	3.8
CBV [ml/100 g]	1.6	1.3	1.3
MTT [s]	33.2	13.4	20.9
Tmax [s]	19.6	10.7	16.5
T0 [s]	3	4	6
W [s]	18	4	4
E	0.400	0.382	0.771

Note that MTT and W estimated by dCTP and mCTA-P were shorter than that of CTP as expected when using a shorter scan duration; however, Tmax was still greater than the diagnostic thresholds used to detect ischemia. CTP indicates CT perfusion; dCTP, down-sampled CTP; mCTA-P, multiphase CT angiography-perfusion; CBF, cerebral blood flow; CBV, cerebral blood volume; MTT, mean transit time; T0, time delay; W, minimum transit time; E, fraction of contrast with transit time greater than W

Supplemental References

1. Meier P, Zierler KL. *On the Theory of the Indicator-Dilution Method for Measurement of Blood Flow and Volume*. *J Appl Physiol*. 1954;6(12):731-744. doi:10.1152/jappl.1954.6.12.731

2. Kudo K, Christensen S, Sasaki M, et al. Accuracy and Reliability Assessment of CT and MR Perfusion Analysis Software Using a Digital Phantom. *Radiology*. 2013;267(1):201-211. doi:10.1148/radiol.12112618

3. Chung KJ, De Sarno D, Lee TY. CT perfusion stroke lesion threshold calibration between deconvolution algorithms. *medRxiv*. Published online November 18, 2022. doi:10.1101/2022.11.17.22282418
